# Transferring an extremely premature infant to an extra-uterine life support system: a prospective view on the obstetric procedure

**DOI:** 10.3389/fped.2024.1360111

**Published:** 2024-02-15

**Authors:** Juliette S. van Haren, Frank L. M. Delbressine, Mark Schoberer, Arjan B. te Pas, Judith O. E. H. van Laar, S. Guid Oei, M. Beatrijs van der Hout-van der Jagt

**Affiliations:** ^1^ Department of Industrial Design, Eindhoven University of Technology, Eindhoven, Netherlands; ^2^ Obstetrics and Gynaecology, Máxima Medical Centre, Veldhoven, Netherlands; ^3^ Institute for Applied Medical Engineering and Clinic for Neonatology, University Hospital Aachen, Aachen, Germany; ^4^ Department of Pediatrics, Leiden University Medical Center, Leiden, Netherlands; ^5^ Department of Electrical Engineering, Eindhoven University of Technology, Eindhoven, Netherlands; ^6^ Department of Biomedical Engineering, Eindhoven University of Technology, Eindhoven, Netherlands

**Keywords:** extra-uterine life support, perinatal life support, artificial womb, artificial placenta, APAW, transfer procedure, neonatal transition, fetal physiology

## Abstract

To improve care for extremely premature infants, the development of an extrauterine environment for newborn development is being researched, known as Artificial Placenta and Artificial Womb (APAW) technology. APAW facilitates extended development in a liquid-filled incubator with oxygen and nutrient supply through an oxygenator connected to the umbilical vessels. This setup is intended to provide the optimal environment for further development, allowing further lung maturation by delaying gas exposure to oxygen. This innovative treatment necessitates interventions in obstetric procedures to transfer an infant from the native to an artificial womb, while preventing fetal-to-neonatal transition. In this narrative review we analyze relevant fetal physiology literature, provide an overview of insights from APAW studies, and identify considerations for the obstetric procedure from the native uterus to an APAW system. Lastly, this review provides suggestions to improve sterility, fetal and maternal well-being, and the prevention of neonatal transition.

## 1 Introduction

Current care for extremely premature infants (<28 weeks of gestational age) involves intensive care, including mechanical ventilation and gastrointestinal nutrition support. Due to their fragile and immature organs coupled with the limits of mechanical ventilation, these patients' mortality and morbidity rates remain high (1, 2). The development of extracorporeal life support to prolong infant organ maturation in a womb-like environment, the artificial womb (AW), has been studied since the 1950s (3, 4). This involves maintaining the infant in a fetal physiological state [i.e., as a perinate (5)] with the lungs submerged in amniotic fluid and maintaining a fetal blood circuit through the umbilical vessels via an artificial placenta (AP). Progress on the development of a fluid environment, maintenance of fetal circulation, and physiological placental blood flow have all led to raised hopes that clinical application has come within reach (3, 6–12). Now that human trials are under consideration (12, 13), the methods used in animal studies must be translated to suit human patients. To date, most experimental research on the technology (except Chamberlain (14) and Westin (15)) has been performed on animal models, which are crucial for the translation of the technology to human patients; however, translational success is uncertain (16). The focus has been predominantly on the critical requirements for continuing fetal homeostasis, meaning sustaining fetal animals for an extended period on external life support by developing and controlling key cardiovascular needs in a liquid environment. Future treatment using such a system also requires changes in the preterm birth care procedure, as the infant would need to be cannulated to an oxygenator and placed in an AW instead of a neonatal incubator. In animal studies, transfer to the AW has been controlled using medication and sedation. In case clinical trials demonstrate improvements in care compared with conventional neonatal incubators, it could significantly impact how obstetric and neonatal care is provided to extremely premature infants.

In this review, we analyze the factors that must be considered from an obstetric standpoint. We emphasize the need for studies on the intervention of fetal-to-neonatal transition within an AW context and provide an overview of the relevant transitional physiology, as it applies to various obstetric approaches. We mapped the key aspects of perinatal physiological changes and reflected on how certain measures should be in place when transferring a perinate to an AW. Second, we analyzed current AW studies and patents, and provided an overview of the transfer methods used. We conclude with considerations and research directions for obstetric procedure development and propose to supplement preclinical investigations with simulation technologies to build further knowledge.

## 2 Data sources

Insights on the physiology of a fetus at birth were gathered through a literature search that informed us about specific milestones requiring intervention, and ideas on how these might be prevented or suppressed. A PubMed search was performed to identify relevant studies on the obstetric considerations. Finally, a manual search of the most relevant literature was conducted. We used a value-sensitive design approach, in which the involvement of a range of stakeholders is emphasized throughout the research (5, 17). This study held expert meetings with medical and technical specialists from obstetrics, gynecology, neonatology, anesthesiology, technical medicine, industrial design, medical engineering, and patient advocates to receive recommendations for the requirements and obtain insights for developing a step-by-step transfer workflow.

Regarding the review of different transfer strategies, since there are only a few published concepts, a manual method was chosen by utilizing PubMed and Google Patents, with search terms written in different combinations of either [artificial AND (placenta OR womb OR uterus OR amnion OR amniotic sac)], [(fetal OR fetus OR preterm OR perinatal OR premature OR infant) AND (extracorporeal OR extrauterine) AND (support OR life support OR environment)]. English articles and patent records were carefully examined to determine whether a procedure describing the transfer of a fetus from a natural womb to an artificial womb was mentioned. First, titles and abstracts were examined from the literature and patent searches to select those that met the selection criteria, followed by full-text screening for the final selection. The reference lists of the included reports were also examined for other relevant articles. For each study, we extracted information on the type of system, animal model, mode of delivery, fetal/maternal medication used during delivery, and a description of the transfer stage. The selection of the studies and patents is presented in Table 1. Not all the published approaches are equal regarding success (e.g., duration of support), validation, research stage (conceptual or experimental), model (e.g., sheep, pig), gestational age, approach (LFL or LFC) and therefore may not be directly comparable or translatable to human application. However, it does provide an overview and demonstrates how knowledge on perinatal physiology is applied in an APAW context.

**Table 1:** List of APAW studies and patents that describe a transfer stage.

Research group	Publications (type)	LFC/LFL	Model	VB/CS	Max. support (hrs)	Fetal/maternal medication given during delivery	Transfer stage description
Philadelphia, USA	2019 (18), 2018 (19), 2017 (3)	LFC	Sheep	CS	672	Buprenorphine. General anesthesia (ketamine, isoflurane, propofol)	Lower midline laparotomy, small hysterotomy made to expose fetus. UC cannulation transitioned to biobag via open sealable side and transferred to the mobile platform.
	2020 (patent) (20)	LFC	–	CS	–	N/A	Maternal hysterotomy to expose fetus, connection to oxygenator, fetus placed on AP circuit. At full circuit flow, the fetus is removed from the uterus and immersed in the fluid-filled incubator.
Toronto, CA/Adelaide, AU	2022 (7), 2021 (21)	LFC	Pig	CS	177	General anesthesia (ketamine hydrochloride, acepromazine, isoflurane, atropine sulfate)	The fetus is delivered while minimizing torsion, stretching, and vasospasm of UC. Normothermia is maintained by continuously bathing the fetus and UC in warmed saline. Following cannulation, fetal pigs were enclosed in a biobag.
	2021 (22)	LFL	Pig	CS		Isoflurane, ketamine and intubated	The fetus was partially exteriorized for surgical procedures; the “trachea piglet” was occluded using surgical ligatures to prevent lung inflation. Fetal piglet exteriorized from the uterusa, placed in a plastic bag for maintenance temperature, and surrounded by warmed heat bags. The UC protruded from the bag, and care was taken to avoid compromise of umbilical blood flow (stretching/kinking). The fetus was immediately placed on a custom-built heated cradle after cannulation after UC clamping and cutting to sustain normothermia.
Michigan, USA	2020 (8), 2019 (23), 2015 (24)	LFL	Sheep	CS	408	General anesthesia. Atropine, buprenorphine, papaverine, lidocaine.	Laparotomy to expose the uterus. Injection into the fetal lamb. Lamb partially delivered, remains supported by the native placenta. At sufficient AP circuit flow, UC is cut, lamb delivered, placed on full AP support with liquid-filled ETT (or MV (1 h) prior to a liquid-filled ETT (23)).
	2013 (25)	LFL	Sheep	CS	70	Buprenorphine, local lidocaine infusion. General endotracheal anesthesia.	The fetus was cannulated and intubated with cuffed ETT. After suctioning airways, lambs were transitioned to gas breathing. Upon respiratory failure, ETT was filled with artificial amniotic fluid, and total AP support was initiated.
	2012 (26), 2009 (27)	LFC	Sheep	CS	24	General anesthesia (sodium thiopental, isoflurane), medication: atropine, buprenorphine, local lidocaine infusion.	Small hysterotomy to expose the fetal neck. A second, small hysterotomy to expose UC at the entry point into the fetus. The fetus is connected to AP. Lambs were maintained on AP support in the natural uterus for 4 h for stability and then transferred to a warmed liquid bath.
Perth/Sendai, AU/JPN	2020 (6), 2019 (28), 2017 (4), 2017 (29), 2016 (30), 2015 (31), 2012 (32)	LFC	Sheep	CS	168	Anesthesia, intubation, ventilation, and premedication: Buprenorphine, phenobarbitone, acepromazine, buprenorphine, midazolam, ketamine, isoflurane, meropenem	Cannulation procedure performed prior to delivery to prevent dehydration. The lambs were covered with warmed saline-soaked sterilized towel. Fetuses were transferred to a liquid incubator, with the bag being promptly filled with artificial amniotic fluid.
Eixarch et al.	2023 (9)	LFC	Sheep	CS	168	Intramuscular fetal anesthesia (fentanyl, midazolam, rocuronium). General anesthesia (acepromazine, ketamine, midazolam, buprenorphine, isoflurane)	Incision to expose the uterus and perform an ultrasound. Hysterotomy to expose UC, before fetal manipulation, fetal anesthesia was administered. After cannulation, the fetus was transferred to a reservoir.
Pak et al.	2002 (33)	LFC	Goat	CS	34	Premedication, general anesthesia (rompun, halothane)	A small hysterotomy to extract fetal hind limbs and expose UC completely. UC connected to AP. UC cut and fetus transferred to a liquid chamber.
Tokyo, JPN	1993 (34), 1990 (35), 1989 (36), 1987 (37)	LFC	Goat	CS	542	Fetal movement suppressor. General anesthesia (halothane)	Incision to extract hind limbs, fetal body was exteriorized until UC appeared completely. After cannulation, UC was cut, and the fetus moved into a liquid incubator.
Sakata et al.	1998 (38)	LFC	Goat	CS	237	Papaverine. Premedication, anesthesia, intubation (atropine sulfate, halothane, nitrous oxide)	After cannulation, the fetus was isolated from the placenta and transferred to a liquid-filled incubator.
Zapol et al.	1969 (39)	LFC	Sheep	CS	55	Spinal anesthesia	After cannulation, the fetus was transferred to a liquid-filled bath.
Alexander et al.	1968 (40), 1964 (41)	LFC	Sheep	CS	24	Anesthesia (procaine, iv sodium thiopentone)	Respiratory movements suppressed by delivering the fetal head into a liquid filled polythene bag.
Chamberlain	1968 (14)	LFC	Rabbit, Human	CS	5	N/A	Amniotic sac removed intact from the uterus, or in case of membrane rupture, fetus placed in a warmed saline at operating table to prevent respiration.
Lawn et al.	1962 (42)	LFC	Pig	CS	7	Anesthesia	Fetuses were set up directly after being taken from uterus. After cannulation the container was filled with solution.
Westin et al.	1958 (15)	LFC	Human	VB	12	N/A	Spontaneous or induced abortion. Cannulation of UC vessels, fetus was placed on a perforated disc above heating apparatus. The chamber was filled with 25 °C liquid to decrease fetal temperature and reduce oxygen consumption. After establishment of circuit, air was released from chamber and closed.
Tchirikov	2017 (patent) (43)	LFC	–	N/A	–	N/A	The transfer of the infant takes place by detachment of native placenta from UC and immediatly embedding into a liquid chamber.
Cooper	2004 (patent) (44)	LFC	–	Both	–	N/A	Fetal mouth is covered.
Greenberg	1954 (patent) (45)	LFC	–	N/A	–	N/A	N/A

LFC, liquid filled chamber; LFL, liquid filled lungs; VB, vaginal birth; CS, cesarean section; UC, umbilical cord; ETT, endotracheal tube; MV, mechanical ventilation; AP, artificial placenta.

Much of our understanding of physiological adaptation in the neonatal transition is derived from animal research and is essential for progress in the field. However, lambs at the same developmental stage as extremely premature infants are significantly larger, pigs are too mature, and non-human primates are too small. Therefore, human trials would require additional adjustments to the APAW setup. These studies’ results and surgical approaches must be considered from the perspective of anatomical, physiological, and developmental differences.

Different terminologies of this particular extracorporeal life support for premature infants can be found in the literature: artiﬁcial womb technology, Artificial Placenta and Artificial Womb technology (APAW) or the system names EXTrauterine Environment for Neonatal Development therapy (EXTEND) (46), ex vivo uterine environment therapy (EVE) (28), biobag, and Perinatal Life Support (PLS) (5). In this review, we refer to APAW and cite published reports using either a liquid-filled chamber or a liquid-filled lung setup.

## 3 Discussion

Normal lung development relies on the womb's liquid environment, adequate intrathoracic space, and regulated intrabronchial pressures (47–49). When born at 24 weeks of gestational age, the preterm lung is in the late canicular stage, when alveolar and capillary development begins (50). Normal alveolar development can be disrupted by preterm pulmonary gas exchange, leading to respiratory failure. Using mechanical ventilation, high volume, and pressure can lead to pulmonary and cerebral injury (50–52). By eliminating the need for pulmonary gas exchange using an AP, retaining liquid within the airways serves a dual purpose: it may avoid fetal to neonatal transition, prevent ventilation and oxygen induced injury, and allow continued maturation of the respiratory system.

The APAW system prevents the physiological transition from fetal to neonatal physiology, normally following birth (53–55). The transition from intra- to extra-uterine life is marked by a series of large and abrupt physiological events. Predominantly triggered by lung liquid clearance and aeration, these events lead to a decrease in pulmonary vascular resistance and an increase in pulmonary blood flow. This causes a shift from fetal circulation with placental oxygenation to neonatal circulation with oxygenation through the lungs (53–55). The decrease in pulmonary vascular resistance and cord clamping initiates a sequence of changes that dramatically reorganizes the infant's cardiovascular system (56–58).

Prostaglandins from the placenta, and adenosine from the liver and placenta, may suppress fetal breathing movements. Adamson et al. observed in experiments conducted in intubated and oxygenated lambs that, when the umbilical cord is clamped, fetal sheep start continuous breathing movements and stop when the occlusion is lifted (59). In newborns, breathing suppression can be reversed by treatment with prostaglandin synthetase inhibitors such as indomethacin (58).

The precise initiation of large inspiratory efforts at birth is not fully understood. However, factors such as activation of chemoreceptors, increased PaCO2 levels, prostaglandins and prostaglandin synthetase inhibitors, loss of inhibitory factors on respiratory center activity and physical stimuli (light, temperature and handling) are thought to contribute (58, 60–67).

If the cord is clamped and fetal-placental circulation is ceased before lung aeration, a sudden 30%–50% loss of venous return from the placenta occurs, with an increase in systemic vascular resistance, as demonstrated in fetal lambs (68).

This transition to neonatal physiology in extremely premature infants occurs too early, as their organs are activated before reaching full maturity and are probably functionally and structurally immature (69). Additionally, current treatments involving mechanical ventilation, although necessary, negatively affect normal growth and development (70). Preventing the transition process and allowing the lungs to achieve full development and maturity may be accomplished by maintaining the liquid in the lungs, thereby preventing the aeration-induced cardiovascular transition (54, 56, 71).

The lungs develop as a liquid-filled organ throughout fetal life, with liquid formed by the epithelial cells of the distal airways. The liquid produced in the airways is causing an intraluminal positive pressure, which is a stimulant for lung development (47, 71). Physiological processes during the last weeks of a full-term pregnancy and the onset of spontaneous labor appear to occur simultaneously with changes in fetal and maternal hormonal balance, preparing the fetus for neonatal transition (72, 73). Normally at birth, lung liquid is replaced by air during the first breaths with the rapid movement of liquid through the epithelium into the interstitial space. Studies in rabbits demonstrated nearly complete clearance within 3–5 breaths across the epithelium, followed by absorption by lymphatic and vessels over 4–6 h (63, 74). During a preterm non-spontaneous birth, such as an emergency preterm cesarean section (CS), preparations through stress hormones (activating epithelial sodium channels and reversing the flux of liquid) and mechanical changes (liquid clearance through uterine contractions) (75) would not have begun, disrupting neonatal transition (76). This results in absent liquid clearance antenatally, leading to more lung liquid volume at birth (77). This delay could be advantageous in preventing lung aeration when transferring to an APAW system.

Full immersion in amniotic fluid enables the fetus to maintain balanced lung liquid (swallowing and absorption), supports gut maturation, and shields against external hazards such as temperature variations, sound, trauma, and pathogens. Whether immediate immersion (with no exposure to air) was performed in the included APAW studies and patents was not clearly defined for each case. In experiments of the Philadelphia group, gas was not allowed to enter the lung (78). Flake et al. developed a biobag with a sealable opening, in which the fetus could be placed after cannulation. At stable perfusion, the opening can be sealed and the biobag can be placed in the support system (20). Flake et al. mentioned that direct delivery from the natural uterus to the liquid filled chamber (LFC) would be ideal; however, more infants could qualify for the procedure if they were supported for some time before moving into the LFC (20).

In an APAW study as early as 1968 (14), respiration prevention was deemed crucial, and infants were kept submerged by an “en caul” delivery. Experiments were performed to understand fetal respiration movements when submerged in a tank and demonstrated that once the cardiovascular circuit was halted, respiratory efforts became more prominent (14). In the study by Alexander, lambs were delivered with their heads in liquid-filled polythene bags to suppress respiration (41).

Harned et al. (79) showed that introducing (amniotic) fluid in the laryngeal region in delivered lambs can result in respiratory suppression, as a correlation between the frequency of swallowing liquid and suppression of breathing was found. Lambs who were still on placental support and had no air exposure demonstrated a possible correlation. While 3-month-old lambs showed no signs of respiratory suppression (79).

Instead of immersing the infant entirely in a liquid-filled chamber, another approach is to occlude the trachea via intubation to maintain the liquid-filled-lungs (LFL). Although direct comparisons with LFC studies cannot be made; both methods can provide insights into how perinatal physiology is modulated in extracorporeal life support. The LFL approach has been demonstrated in several animal studies via CS, after which the fetus is injected with sedatives and the umbilical vessels are cannulated. Once a stable circuit exists, the fetus is fully exteriorized and an endotracheal tube is introduced (8, 24, 25). In this approach, fluid with oral chlorhexidine is supplied to the endotracheal tube (ETT) to reduce infection, which is subsequently occluded. Daily ETT insertions of additional amniotic fluid were performed to maintain a constant fluid level without pressurization (24, 25). In another study, perfluorocarbon was used instead and maintained at a set pressure in a closed system (8). Obstruction of the fetal trachea could cause overexpansion of the lung, making it necessary to allow normal fluctuations in intrabronchial pressure and liquid breathing movements to occur.

Several APAW experiments have used medication to suppress fetal (breathing) movements or general anesthesia to control breathing (8, 24, 34–26). If medication to suppress respiration were to be used in humans, it would be ethical to combine breathing suppressants with adequate sedation. Selecting appropriate sedatives and exposure duration is crucial to avoid the risks of adverse neurodevelopmental effects, gastrointestinal motility issues, and lowered arterial blood pressure (80, 81).

Other methods to **suppress air-based respiration** also need to be considered. In addition, it might not always be possible to have the APAW system and necessary neonatal intensive care staff members present during delivery while in the operating room. Moving the preterm infant to the neonatal ward should therefore ideally be performed while the lungs are submerged in liquid.

Potentially harmful effects of preterm lung aeration, like oxygen toxicity or termination of the fetal circulation while connected to an artificial placenta, suggest that lung aeration during the transfer procedure ought to be avoided. Normally, during the first breathing, the air is inhaled into the (surfactant-rich) mature lungs. Yet, the underdeveloped lungs of extremely premature infants often struggle to effectively aerate the lungs, necessitating some form of respiratory assistance (63). This difficulty may be attributed to factors such as the structural immaturity of the lungs, inadequate respiratory drive, impaired lung liquid clearance, muscular weakness, rib flexibility, and surfactant deficiency (82). This suggests that limited gas intake has a small effect. As oxygenation of the perinate would be ensured via the artificial placenta, the cardiovascular effects are thought to remain unchanged. More research is necessary to elucidate the underlying physiological mechanisms, understanding the associated risks and the potential reversibility by introducing lung liquid, as demonstrated in animal experiments by Gray et al. (25). The Michigan group showcased that lambs could initially receive mechanical gas ventilation, but when respiration failed (57 ± 22 min), they could successfully be transferred to breathing liquid by filling the endotracheal tube with artificial amniotic fluid (25). This raises questions about short-term air intake effects on triggering inflammatory cascades and causing lung injury, the effect on the cardiovascular circuit (e.g., the ductus arteriosus), and whether a transition to neonatal physiology has been initiated and/or whether this transition is reversible. Postmortem analysis confirmed patent ductus arteriosus, foramen ovale, and sinus venosus in all subjects (25). The specific limits of gas breathing determining if small periods of breathing gas during the canalicular phase, may cause lung injury are yet to be determined (83). More (preclinical animal) studies are needed to evaluate functional and structural lung damage, both from lung aeration and efforts to prevent it. Thus, based on theoretical considerations, preventing gas from entering the lungs completely and preserving airway liquid in an APAW context appears desirable. In current care, when at risk of preterm delivery, the mother receives corticosteroids (i.e., betamethasone) to increase lung maturation, thereby improving neonatal outcomes, such as reduction of germinal matrix and intraventricular hemorrhage (84, 85). Further research is needed to investigate and clarify the sequential cascade of effects of antenatal corticosteroids. As the aim of APAW treatment is to maintain fetal physiology, preventing pulmonary gas exchange, further research is necessary to understand whether administering corticosteroids should be standard when transferring to APAW.

*1. During the transfer procedure, exposure of the perinate's airways can initiate the breathing reflex. Based on the theoretical insights, it appears logical to prevent this by maintaining liquid in the lungs of infants.*

The infant leaves the warm uterus environment and enters the cold hospital room with an ambient temperature of 26°C to 27°C (86). Within several seconds after birth, the **infant's temperature** decreases by approximately 2°C (87). Fetal temperature is on average 0.3°C to 0.5°C higher than the maternal temperature (86), with mean measurements ranging from 37.1°C at the beginning of labor and increasing to 37.4°C after 22 h (88). A fetal temperature exceeding 41.0°C should be avoided due to risks of protein denaturation in the fetal brain (89).

Preventing hypothermia has become standard care during preterm delivery (90). Because preterm infants have a high surface-area-to-volume ratio and low subcutaneous fat content, they quickly lose body heat (91).

In nearly all APAW studies included, the temperature of the infant was kept in line with in-uterus values (ca. 39.0°C) (11). However, one of the first studies performed decided to keep the fetus at a colder temperature before cannulation to keep the oxygen consumption low (15). After perfusion was established, the infant was placed in warm liquid. Under these conditions, no respiratory movement was observed (15).

*2. The liquid environment in which the perinate would be captured should be temperature-controlled to prevent the child from developing hypo- or hyperthermia* (89, 91).

A crucial task during transfer is **cannulation of the umbilical vessels**, thus connecting the umbilical cord to the artificial placenta (11).

Sobotka et al. investigated the degree of heart rate decline upon umbilical cord occlusion in ex-utero and in-utero lambs (92). They suggested that a newborn's immediate environment substantially impacts cardiovascular responses to prenatal hypoxia. They theorized that this may be influenced by the “diving reflex”, which is a vasovagal reaction brought on by contact with water on the face (92, 93). Chamberlain showed that when a fetus is placed in liquid, and the umbilical cord is occluded, gasping movements increase, which is an effort to maintain O2 levels (14).

Maintaining standard placental blood flow is essential for adequate fetal oxygen delivery at normal saturation levels. A single 10-min occlusion of the umbilical cord causes neuronal loss in (predominantly) the hippocampus, as demonstrated in fetal sheep (94–96). Within approximately 4 min of total cord occlusion in fetal sheep, a loss of cerebral blood flow autoregulation can be expected (97).

Although no exact duration of safe cord occlusion can be determined in humans, compression or overstretching of the umbilical cord regularly occurs during normal delivery, resulting in temporary fetal bradycardia as a physiologic response to the hypoxemic state.

Recent studies used sheep models to demonstrate rapid vessel cannulation. Partridge et al. also observed unconstrained fluid breathing, and swallowing movements while ensuring a rapid cannulation setup to ensure timely oxygen delivery for brain oxygenation (3). However, it is important to note that the sheep umbilical cord anatomy, comprising two umbilical arteries and two umbilical veins, enables the support of the native placenta through the second set of vessels while shifting to AP support. In humans, the single umbilical vein does not allow continued native placental support, making studies in pig animal models closer to human translation (22). More research is needed to draw requirements for human patient umbilical vessel cannulation (98).

Studies have performed vessel catheterization in animals *in utero* or ex utero. In cases of exteriorization before cannulation, the animal was generally placed in a bath, bag, or kept warm. Due to the length of the umbilical cord at 24 weeks' gestational age, cannulation after delivery would need to occur in proximity to the mother.

Knowledge on cannulation strategies can be gained from existing ex utero intrapartum treatment (EXIT), such as those described for EXIT-to-extracorporeal membrane oxygenation (ECMO) procedures (99) and could aid in determining a safe sequence of cannulation. Next to avoiding fetal airway exposure to air, factors such as uterus involution, shear-stress within the utero-placental unit and umbilical cord length, may also need to be considered to determine the optimal position of the fetus during cannulation.

*3. Rapid establishment of the artificial placenta circuit is required to maintain arterial pO2 and pCO2* (92).

The supply of oxygen-rich blood from the placenta to the fetus is dependent on patency of the ductus arteriosus. This vascular shunt connects the main pulmonary artery to the aorta (100, 101). This shunt must remain open during pregnancy and birth to bypass the amniotic-fluid-filled fetal lung. Fetal adaptation after delivery necessitates the emergence of spontaneous breathing and increased oxygen tension, which leads to ductus arteriosus closure (102, 103). Although the exact processes regulating ductal patency and closure are unknown, placental prostaglandins (PG) are involved (100, 101). PGE1 and PGE2 keep the ductus arteriosus open (104). Normally, at birth, when the fetus separates from the placenta, the amount of vasodilatory placental PGs in circulation decreases (103). Neonates born with a patent ductus arteriosus can therefore be administered PGSI's (such as indomethacin) (105). PGE1 was administered to lambs in a study by Partridge et al. (3), presumably to maintain patent ductus arteriosus. PGE1 and synthetic PGE1 (misoprostol) are routinely used in clinical practice to induce labor and enhance circulation in neonates with ductal-dependent cardiac lesions before surgery (104, 106). To maintain the fetal cardiovascular circuit and prevent fetal-to-neonatal transition, closure of fetal shunts (such as the ductus arteriosus) needs to be prevented (54). To confirm persistence of the fetal cardiac circulation, previous APAW studies in animals have taken echocardiographs daily, to verify the opening of the ductus arteriosus, ductus venosus, and foramen ovale (3, 4). To ensure fetal cardiac circulation, hormones (such as PGE1) could be administered within the APAW system to prevent shunt closures.

During fetal development, endogenous glucocorticoids (cortisol and corticosterone) are crucial in organ maturation, such as the brain, lungs, kidneys, liver, and thyroid (107). At term, preparation for birth, transition to neonatal physiology, and multiorgan adaptation involve increased catecholamines and cortisol release (58). Administering exogenous glucocorticoids (dexamethasone or betamethasone) to women at risk of preterm labor accelerates fetal maturation—such as stimulation of surfactant production, thereby improving newborn outcomes (108, 109). However, excessive or premature exposure of the fetus to glucocorticoids may disrupt developmental pathways, resulting in disrupted growth, impaired gas exchange, and potential harmful long-term changes in physiological function (107, 108, 110). The fetal hypothalamic–pituitary–adrenal axis is especially prone to glucocorticoid-induced changes with potential long-lasting effects (107).

Small-for-gestational-age fetuses already have high endogenous cortisol levels; therefore, exogenous antenatal corticosteroids before preterm delivery may not provide advantages (110). In growth-restricted fetuses, antenatal exposure to high levels of endogenous glucocorticoids are thought to affect the regulation of cardiovascular development. These infants rely more on the sympathetic nervous system to maintain blood pressure and redistribute cardiac output for vital organ growth, which could be further impaired by exogenous glucocorticoids (111, 112).

Similarly, the use of antenatal dexamethasone to enhance fetal maturation has been associated with reduced birth weight (108). However, antenatal corticosteroids have decreased newborn mortality without affecting morbidity in preterm small-for-gestational-age infants (110).

The impairment in adaptation observed in extremely premature infants and extending fetal physiology using APAW require further investigation to determine whether the suppression or acceleration of neonatal adaptation is appropriate.

Within the context of an APAW system and the maintenance of fetal physiology, the promotion of lung maturation would not be suitable. However, the decision to withhold or administer antenatal corticosteroids is complex given the possibility of treatment redirection to conventional care (rescue procedure) and the uncertainty surrounding the optimal timing of delivery for a preterm infant (113). If a transfer procedure reverts to standard care, preterm care must align with conventional care standards and outcomes, and therefore inform the decision on altering antenatal administrations.

Certain antenatal administrations, such as magnesium sulfate, have shown neuroprotective effects for preterm births, and it would be a reasonable question of research, if this also applies to patients in an APAW context, seen that extremely preterm infants are at increased risk of neurologic injury (114, 115).

To sum up, the application of standard-of-care **antenatal pharmacological interventions** should be further investigated in cases of APAW treatment.

*4. Medication may be necessary before and during transfer to prevent specific physiological cascades related to neonatal physiological transitions, for their (neuro)protective effects and to support organ growth and maturation. Further investigation is required to determine the suitability of administering conventional medications, such as corticosteroids*.

Another essential factor for the successful transfer of the perinate is **intact umbilical and placental circulation**. When blood flow in the umbilical cord is reduced the same holds for oxygen consumption, as shown in fetal lambs (116), which could lead to transfer failure and health risks to the perinate. Umbilical cord obstruction can be caused by the pressure exerted by a physician (leading to hematoma), vasospasm, and interaction with surgical tools and actions. Additionally, the occlusion of the umbilical cord stimulates breathing initiation regardless of blood gases and pH, as demonstrated in fetal sheep (61). When occluded, the pulmonary stretch receptor activity first increases and subsequently decreases to zero at approximately the same time as the breathing reflex (117).

*5. The transfer procedure should avoid obstruction or damage of the umbilical cord that could lead to the occlusion of the umbilical cord's blood flow*.

Ideally, transfer to the APAW system could be performed via **vaginal and CS delivery**. Planned CS is always performed before vaginal delivery could occur, thereby increasing the risk of preterm birth. Delaying delivery, by performing vaginal delivery in certain situations could impact survival in cases of extremely premature birth (118). Additionally, preterm birth of an infant cannot always be planned; therefore, the timing does not always allow for CS. A vaginal procedure is preferred for the mother, as additional CS delivery risks, such as incision and uterine infection, pulmonary embolism, increased blood loss, placental growth into the scar in subsequent pregnancies, and uterine rupture, can be avoided (118, 119). Nevertheless, a transfer via vaginal delivery following preterm premature rupture of membranes may affect the feasibility of potential APAW treatments. Potential beneficial effects may be found for patients with lung hypoplasia after re-immersion into an adequate amount of amniotic fluid (120). Nevertheless, research attention should be given to maintaining the sterility of the environment, especially since chorioamnionitis is responsible for triggering roughly half of preterm births (121). An association has been found between chorioamnionitis, or early onset bacterial infection, and an increased risk for germinal matrix intraventricular hemorrhage (122, 123), a risk that has already shown to be increased in ECMO treatment in these infants (124).

To increase the success rate for a perinatal transfer, and because nearly half of premature births are performed via CS, a CS transfer procedure should also be available (125–128). From a fetal perspective, CS transfer offers a more controllable environment throughout the procedure. In contrast to vaginal birth, in a CS the “labor less” placenta should function similarly as during pregnancy, provided specific anesthesia protocols are followed. Relevant surgery has been successfully applied to the human fetus in this condition, i.e., ex utero intrapartum treatment, whilst keeping the fetus on uteroplacental circulation (129).

In a broader context, the delivery mode can impact the transition stage to neonatal physiology. Certain processes involved in neonatal transition leading to spontaneous vaginal labor would be omitted if elective CS is planned before labor begins. Therefore, CS can lead to retained lung fluid and (transient) poor respiratory adaptation (58, 128). However, because the transition is impaired in extreme preterm births, a difference in the stage of neonatal transition between CS and vaginal delivery is less likely.

Previous APAW animal studies controlled umbilical vessel cannulation directly after CS while the animal was still *in utero* or ex utero, connected to the native placenta, before vasospasm or cord desiccation could occur (Table 1).

Through the CS and a small hysterotomy, the fetus is exposed and can be cannulated in the neck or umbilical cord. After establishing the circuit, the fetus was transferred to a warmed fluidic incubator. A similar approach was demonstrated by the Philadelphia group, where a lamb was placed directly in a biobag after delivery and cannulation, after which the biobag was sealed and transported to a mobile support station (3). They proposed the possibility of vaginal delivery by cannulating at the perineum (12). In contrast to the broader applicability of AP studies in terms of delivery modes, current EXTEND protocols continue to focus on CS (78).

One of the first approaches described for human subjects was to perform CS and keep the native amniotic sac intact, after which the fetus could be submerged in the artificial amniotic fluid in a tank. Subsequently, cannulation was performed. In case of membrane rupture, the fetus is placed in a warmed fluid on the operating table (14). In 1958, a study performed in humans reported transfers by vaginal, spontaneous, and legally induced abortions. After delivery, the umbilical cord was clamped rapidly and the infant was kept in a temperature of 25°C, as this is believed to reduce the oxygen consumption until the circuit is established. After umbilical vessel cannulation, the fetus was placed in a closed liquid-filled chamber on a perforated disc above the heating apparatus (15).

In a patent published in 2004, Cooper suggested two possible transfer methods: CS and birth canal (44). As the only detailed account describing a human obstetric procedure, we included it despite a lack of feasibility studies. A CS can be performed without rupturing the membranes, allowing for an “en caul” delivery where the fetus remains protected within the amniotic sac, subsequently placed in a net and submerged in warmed artificial amniotic fluid (44). If membrane rupture occurs, the medical staff should prevent the infant from breathing by covering the infant's mouth. This approach has also been suggested for VB transfer, with the physician's hand in the infant's mouth before placing it in a submerged net. In the case of VB, the contamination risk is a concern, which can be mitigated by cleaning the fetus through successive antibiotic and antimicrobial baths.

Infants from a multiple pregnancy could particularly benefit from improved preterm care, as they currently face a higher likelihood of being born preterm (130) and have a higher risk of adverse outcomes compared to singletons at similar gestational age (131). Factors to address in future research on this topic may include management of umbilical cord abnormalities and cannulation strategies in twins.

*6. Efforts should be made to allow the transfer procedure to be available for both vaginal and CS delivery, and to allow for a multiple birth*.

Vaginal delivery is not sterile, exposing the fetus to the mother's vaginal flora during birth. In addition, the uterus is not a sterile environment, as studies have shown that the acquisition and colonization of the human digestive tract begins *in utero* (132). In extremely preterm births, chorioamnionitis is the most common cause of preterm labor (133).

Some studies have compared vaginally delivered neonates exposed to maternal vaginal flora vs. CS-delivered neonates and argued that exposure to vaginal flora may contribute to developing the newborn immune system (134, 135). Therefore, contact with the vaginal microbiota could be explored, for example, during the birth from the APAW system (136). However, no exposure to vaginal flora should occur throughout the present process, because it is intended to maintain perinatal physiological conditions in the fetus. Exposing the perinate to vaginal flora during transfer may increase the risk of Early Onset Sepsis, which is most often acquired from the mother's genital tract or, less frequently, vertically through the placenta (137). Infection with group B Streptococcus is the leading cause of morbidity in Early Onset Sepsis (138). A vaginal swab and potential antibiotic treatment must be given as per standard practice in managing threatened preterm labor.

Although not all studies have demonstrated significant outcomes (139, 140), intrapartum intravaginal lavage can be regarded as a feasible precaution to limit the risk of newborn contamination with pathogens from the maternal genital canal (141). Specifically, chlorhexidine and povidone-iodine (PI) treatments may be beneficial (142, 143). However, data on the possible adverse effects of chlorhexidine exposure in the birth canal of neonates, particularly preterm newborns, are limited. Multiple examples of adverse skin responses to topical chlorhexidine have been reported, with extremely low birth weight neonates suffering the most serious reactions (i.e., burns) (144, 145). This was most likely caused by compromised skin integrity (146). Perinates may also swallow or inhale any residual chlorhexidine present during delivery. However, there is currently no literature on its impact on preterm newborns. A study on rats showed acute pulmonary inflammation, capillary congestion, edema, and interstitial fibrosis after gradual intratracheal administration of 0.1% and 1% chlorhexidine solutions (147). These histological changes are comparable to those that can be seen in patients who suffer from acute respiratory distress syndrome (148–150). The researchers concluded that there is a reasonable chance that inhalation of chlorhexidine at concentrations of 0.1% or greater could result in this syndrome; thus, using chlorhexidine would ideally be avoided.

PI has also been used to disinfect birth canals prior to cesarean section and vaginal delivery and showed reduced infection rates (141, 151). According to one study, infection rates following standard vaginal saline solution disinfection were comparable to those following PI disinfection (152). The same was true for vaginal cleaning using a diluted baby shampoo (153). Additionally, prolonged exposure to PI (1%–2%) during pregnancy or birth may trigger temporary thyroid dysfunction in infants and mothers (154). According to some studies, this condition may be tolerated if the newborn is well monitored after exposure. However, it may be preferable to use only non-iodine antiseptics.

Alternatively, rather than chemical protection, the use of a physical barrier, such as a retractor between the perinate and birth canal, could be investigated to prevent **exposure to vaginal flora**.

Multiple techniques have been proposed to reduce the perinatal contamination in liquid-filled incubators. Sakata et al. added antibiotics to lactated Ringer's solution (38). When the perinate is transferred via the birth canal, more attention should be paid so that the vaginal microflora are not taken into the incubator. Cooper suggested performing subsequent rinses in baths containing antibiotics and antimicrobial fluids (44). Antibiotic treatments (meropenem, fluconazole, and cefazolin) have also been regularly administered intravenously to lambs in liquid-filled incubators to prevent infections (4, 6, 29).

APAW studies in animals have shown that some lambs die of pulmonary inflammation (3, 6). Partridge et al. compared multiple options for fluid chamber design and demonstrated that the use of a sealable biobag instead of open or semi-open structures eliminated many problems related to fluid contamination and associated infections (3). Owing to their system's fluid-filtering capacity, the Philadelphia group suggests that any contamination could be removed after placing the infant in the system (12). Adding antimicrobial coatings to an incubator can also aid in reducing bacterial growth on the film (20, 44).

*7. Ideally, exposure to vaginal flora should be prevented during the perinate transfer. We argue that a physical barrier between the fetus and birth canal will likely prevent or greatly lower fetal exposure to vaginal flora and possible lavage residues*.

For the safety of the perinate, it must be possible to cease transfer to the APAW system at any time during the procedure and proceed with conventional neonatal care. Establishing specific criteria (and parameter thresholds) is essential for determining whether and when a **rescue procedure** is necessary. The critical phase of vital parameter monitoring is the time between the abdominal incision and attachment of the inserted cannulas to the extracorporeal circuit. This could be regarded as the transition time from fetal heart rate monitoring (CTG) to more comprehensive monitoring of the infant, since data on blood pressure, heart rate, and oxygen saturation are immediately available once a circuit is established. However, if the cannulation procedure fails or is prolonged, monitoring relies solely on inobtrusive methods (e.g., CTG) or monitoring would have to be extended to attach sensors to the fetus *in utero*.

A protocol that specifies the maximum duration of cannulation, identifies the key clinical parameters that indicate deterioration, and emphasizes the importance of promptly discontinuing the procedure before the onset of irreversible damage, such as decreased cerebral oxygenation, should be established. Future preclinical studies should identify all the relevant parameters and their threshold values, which could be aided by the development of a clinical decision support system (155). It is essential that these criteria are unambiguous, and that the parties responsible for making the decision to cease the transfer are clearly delineated. These may include parents, obstetricians, neonatologists, and/or technicians.

*8. The primary objective of monitoring during the transfer is to indicate that everything is proceeding as expected or to indicate promptly that the rescue procedure must be started before irreversible harm occurs*.

The effectiveness of prospective APAW treatment should be evaluated based on its improvement over the standard of care for an extremely premature infant in the neonatal incubator on a ventilator, not by comparison with the native womb (156). However, knowledge of the intrauterine conditions should inform APAW treatment to improve preterm care outcomes. General benefits can be gained from further research on the environmental influences that affect development (157). Birth is associated with massive tactile, visual, auditory, and vestibular sensory sensations, when the fetus leaves the warm uterus for a new but cold physical environment. Although no clear conclusions can be drawn from the available research that short-term exposure to the external environment affects perinatal development, **attenuation of environmental triggers** from the harsh hospital room during delivery would allow a smooth transfer from the protective environment of the native womb to the protective environment of the liquid-filled chamber (LFC). Submerging the fetus in a liquid has the benefit of protecting it from sound, balance disturbances, tactile stimuli, and trauma. Additional efforts could include protection from intense light while allowing visual examination by medical staff. For instance, Charest-Pekeski et al. covered biobags to prevent light transmission to the fetus and mimic uterine conditions more accurately (7). While detailing these requirements is beyond the scope of this review, we believe that the obstetric procedure should be in line with the attenuation of triggers offered in an LFC and therefore avoid exposure to temperature shifts, noise, and harsh directed lights.

*9. Despite the transfer taking only a limited duration, it would be optimal to attenuate environmental triggers, as is aimed for in an LFC, yet allow for adequate visual examination of the perinate to make an informed decision to (dis)continue treatment*.

Contributions towards health and well-being that account for the emotional impact on mothers and partners should not be overlooked. Maternal comfort could be facilitated by epidural or spinal analgesia as general anesthesia could carry more risk of maternal and neonatal complications (158–160).

In previous studies on APAW with lambs, ewes were premedicated, anesthetized, intubated, and ventilated by administering drugs including buprenorphine or propofol (3, 28). Buprenorphine, an opioid, depresses ventilation, which is beneficial for suppressing the breathing reflex of the fetus (161, 162). Another opioid that is used in clinics for maternal **pain relief** is pethidine, a morphinomimetic (163). This opioid also suppresses the breathing reflex in the neonate (164). In vaginal births, epidural is the preferred pain relief option, or remifentanil is used as an alternative, which crosses the placenta but is thought to be quickly metabolized. In cesarean sections, spinal anesthesia is preferred. Although fetal surgery can be performed under fetal-direct anesthesia, it is primarily reserved for pain management in critically ill patients and represents a decision between two unfavorable options. If a transfer could be performed without the use of fetal anesthetics (but with maternal anesthetics), it would be more desirable if it did not result in an unreasonable pain experience (165, 166).

*10. With appropriate pain relief in place, attempts should be made to avoid general anesthesia*.

Although the enclosed environment of the LFC is designed to protect the infant from external factors and sustain fetal physiology, it also prevents direct contact, or potential **bonding, between parent and infant**. Studies have shown a correlation between postnatal attachment and maternal and newborn outcomes (167). High levels of bonding are related to greater adjustment to the parental role from the mother, and are positively correlated with social, cognitive, and physical development in the newborn (167–170). Insufficient bonding in extremely premature infants can negatively affect hormonal, epigenetic, and neuronal development (170).

Upon delivery, the mother undergoes significant endocrine changes triggered by the detachment of the placenta (171, 172). These hormonal fluctuations, such as a rapid decline in the concentrations of estradiol and progesterone after birth, could contribute to depression, anxiety, or increased stress levels (172, 173). Preterm delivery can turn into a traumatic experience due to fear, helplessness, pain, and loss of control (174, 175). Quality of care, privacy, engagement in decision-making, and support significantly impact a woman's birth experience (176). Unplanned CS and instrumental vaginal deliveries have been associated with increased risk of negative birth experiences and maternal health issues (177, 178). Early maternal-newborn contact directly following delivery, such as seeing, holding, and feeding, has been linked to positive birth experiences for new mothers, especially those with CS (179). Situations may arise in which patients prepared for, or on APAW support do not survive, and parents may not have had the opportunity to hold or witness their infant alive. Adequate parent support guidelines, such as those that exist for stillbirth, should be in place.

Additional research should confirm the direct connection between negative birth experiences and subsequent poor maternal caregiving (180). In any case, adequate obstetric and family centered care policies are imperative to enhance patient well-being.

Regarding birth experience, the LFL approach would be more similar to the current neonatal intensive care, and skin-to-skin contact between parents and infants would still be possible (181). Further (long-term) research will elucidate which configuration is the safest and most preferable for patients.

*11. Parent-infant bonding (either through direct contact or technology-enhanced bonding) is advisable, as it could enhance the birth experience and, thereby, parental and fetal well-being*.

Finally, integrating treatment using APAW into the current healthcare system will likely demand increased **human and material resources**. Personnel setups required for novel treatments, such as transfer procedures, require specialized training and may easily exceed staffing availability. For example, the health workforce requirements in German perinatal centers demand the presence of 2 gynecologists, one neonatologist (present or on-call), one fellow pediatrician, one anesthesiologist, one scrub nurse, one surgical technical assistant, and one medical assistant (182). Within the context of a transfer procedure, to cover delivery, cannulation, and placement in the LFC, the majority of this team would likely be needed, thereby preventing them from caring for other patients. This makes treatment with this technology highly specialized, expensive, and inaccessible to many patients, as low- and middle-income countries are already struggling with staffing shortages for newborn care (183). Centralizing care may initially be necessary if this specialized treatment is brought to the clinic, requiring the accumulation of experience and expertise, along with the need to rearrange the NICU ward and purchase of medical equipment.

*12. Human and logistical resources should be considered to integrate treatments using APAW within the existing healthcare system*.

Research suggests that medical simulation may improve obstetric team performance and maternal and perinatal outcomes (184). Medical simulation has also been proposed within an APAW context to complement the development of procedures tailored to human patients (5, 155, 185). Although in silico studies should ultimately be followed by validation *in vivo*, the emergence of novel simulation technologies could offer an intermediary step that allows for a controlled and safe environment for procedural development and training. A large body of literature has been written about the ethical development of APAW technology (5), underscoring the crucial role of dialogue and stakeholder engagement during its development.

**Figure 1:**
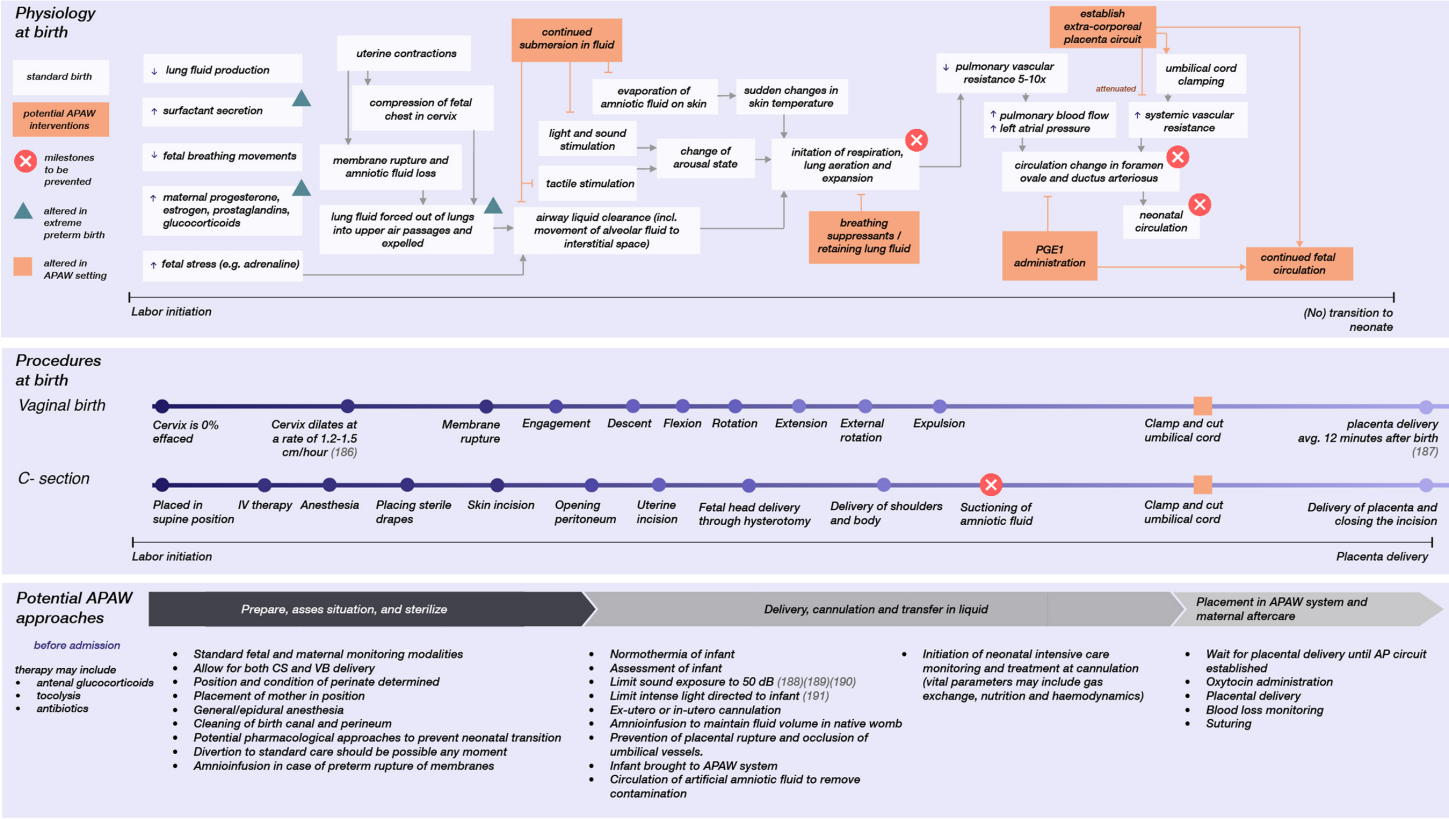
The physiology involved at birth that results in neonatal transition, the different modes of delivery, and potential obstetric approaches for a transfer to an APAW system. Each has a relative timescale (186–191).

Figure 1 summarizes the considerations for a transfer procedure, as it relates to standard obstetric care for extremely premature infants, and lists points for future research that focus on obstetric approaches in an APAW context. Future research on the obstetric approaches for APAW treatment is here divided into three stages surrounding preterm care: pre-, during and post-delivery. Apart from obstetric care, prompt initiation of neonatal intensive care monitoring and treatment at cannulation would be crucial for continuous adjustment of gas exchange, nutrition, medical treatment, stabilization of the circulation, countering extracorporeal circulation side-effects, obtaining blood samples for various tests, and initiating infection control if necessary.

## 4 Conclusion

This narrative review covers the transitional physiology in an APAW context and how it relates to different obstetric approaches and perinatal physiological needs. The majority of APAW studies included in this review, conducted on animals, have been essential in providing valuable insights; however, their applicability in humans remains uncertain. The feasibility of transitioning from placental to extracorporeal circuit support with conventional obstetric care and the associated requirements, such as safety thresholds, remain unclear.

This review aims to outline future research directions and lists considerations for a safe obstetric procedure to mitigate potential risks. This can be achieved by promoting continuous intrauterine conditions through adaptation of conventional preterm delivery. Considerations include preserving fluid in the airways, maintaining sterility, normothermia, anesthetic management, attenuating external stimuli (e.g., light and sound), and ensuring psychological aspects such as patient well-being. Certain requirements may become superfluous as research advances.

Compared to the present preterm care, one challenge associated with the potential clinical use of AW is the necessity of CS—as seen in all successful AW models. Clinical translation may require an EXIT procedure or a modified CS. Vasospasm and reduced placental perfusion at the time of initial vessel cannulation could favor CS delivery over VB, especially in cases of prolonged labor. Still, allowing both vaginal and CS deliveries could benefit maternal outcomes if measures to limit vasospasms, prevent neonatal transition and the promotion of sterility are ensured.

Preclinical studies have shown successful maintenance of APAW support and many learnings can be drawn from its animal studies. As there are still missing links in APAW research that need to be resolved before clinical use (192), animal models can also aid in determining the physiological factors involved in neonatal transition and how they are modulated during extracorporeal life support.

Medical simulation can be complementary to reach this aim, as these can seamlessly integrate a value-sensitive approach to identify and implement patient-centered care and ethical considerations in APAW development, ensuring optimal alignment with the needs and values of stakeholders (5, 17).

In this review, we aimed to provide a comprehensive overview of factors that might come into play in the obstetric care of future treatment using APAW technology and provide suggestions for how these factors might be addressed. Building a collective understanding of essential transitional physiology and obstetric practices informs technological development and can enhance the readiness of a medical team in anticipation of potential future clinical trials.
